# Characterization of Short-Term Heat Stress in Holstein Dairy Cows Using Altered Indicators of Metabolomics, Blood Parameters, Milk MicroRNA-216 and Characteristics

**DOI:** 10.3390/ani11030722

**Published:** 2021-03-06

**Authors:** Jang-Hoon Jo, Jalil Ghassemi Nejad, Dong-Qiao Peng, Hye-Ran Kim, Sang-Ho Kim, Hong-Gu Lee

**Affiliations:** 1 Department of Animal Science and Technology, Sanghuh College of Life Sciences, Konkuk University, Seoul 05029, Korea; godandthegod@naver.com (J.-H.J.); jalilgh@konkuk.ac.kr (J.G.N.); pdg15689@foxmail.com (D.-Q.P.); 2 Animal Nutrition and Physiology Team, National Institute of Animal Science, RDA, Wanju 55365, Korea; ococ1004@korea.kr (H.-R.K.); kims2051@korea.kr (S.-H.K.)

**Keywords:** heat stress characterization, heat stress indicators, lactating Holstein cow, metabolomics

## Abstract

**Simple Summary:**

In this study, we characterize the influence of short-term (4 days) heat stress on Holstein cows during early lactation. The use of indicators, such as production performance, physiological variables, blood parameters, micro-RNA expression, and metabolomes, in heat-stressed cows during early lactation—which is a high-stress phase—may provide insights into how to deal with the level of damage to dairy cows, through appropriate nutritional and management strategies. We identify that short-term heat stress has a negative effect, to some extent, on feed and water intake, rectal temperature, heart rate, blood hematology and metabolites, milk characteristics, miRNA expression in milk, and metabolomics in blood.

**Abstract:**

This study aims to characterize the influence of short-term heat stress (HS; 4 day) in early lactating Holstein dairy cows, in terms of triggering blood metabolomics and parameters, milk yield and composition, and milk microRNA expression. Eight cows (milk yield = 30 ± 1.5 kg/day, parity = 1.09 ± 0.05) were homogeneously housed in environmentally controlled chambers, assigned into two groups with respect to the temperature humidity index (THI) at two distinct levels: approximately ~71 (low-temperature, low-humidity; LTLH) and ~86 (high-temperature, high-humidity; HTHH). Average feed intake (FI) dropped about 10 kg in the HTHH group, compared with the LTLH group (*p* = 0.001), whereas water intake was only numerically higher (*p* = 0.183) in the HTHH group than in the LTLH group. Physiological parameters, including rectal temperature (*p* = 0.001) and heart rate (*p* = 0.038), were significantly higher in the HTHH group than in the LTLH group. Plasma cortisol and haptoglobin were higher (*p* < 0.05) in the HTHH group, compared to the LTLH group. Milk yield, milk fat yield, 3.5% fat-corrected milk (FCM), and energy-corrected milk (ECM) were lower (*p* < 0.05) in the HTHH group than in the LTLH group. Higher relative expression of milk miRNA-216 was observed in the HTHH group (*p* < 0.05). Valine, isoleucine, methionine, phenylalanine, tyrosine, tryptophan, lactic acid, 3-phenylpropionic acid, 1,5-anhydro-D-sorbitol, myo-inositol, and urea were decreased (*p* < 0.05). These results suggest that early lactating cows are more vulnerable to short-term (4 day) high THI levels—that is, HTHH conditions—compared with LTLH, considering the enormous negative effects observed in measured blood metabolomics and parameters, milk yield and compositions, and milk miRNA-216 expression.

## 1. Introduction

Environmentally induced hyperthermia is a global-scale threat to the dairy industry in many ways, with its effects including economic loss, animal health issues, and productivity. The adverse effects of heat stress (HS) on the productivity of dairy animals, in terms of milk yield, composition, and quality, have been well-documented [1]. Despite advances in cooling systems and environmental management [2], HS continues to negatively affect the diversity of dairy production characteristics. As such, lactating dairy cows under HS experience limited energy intake and, thus, are unable to meet the demands of their bodies for maintaining milk production and health, resulting in reduced milk yield and quality and leaving the animals susceptible to diseases [1,3,4]. The harm of HS is particularly more important in high-yield dairy cows, due to their higher susceptibility to stressors which periodically happen during early stage of lactation [5,6,7].

The temperature humidity index (THI) has exclusively been employed as an index of HS status in cattle [3]. However, short-term and long-term exposure to HS may reflect variations in responses of the animals. Furthermore, THI is based only on the air temperature and humidity [3] and, as such, is not a direct biomarker of metabolic alterations in response to HS. Thus, alterations in physiological parameters, such as rectal temperature, heart rate, and respiratory dynamics, reflect the degree of HS in cattle [8], as well as feed intake [1,4], as the first indicator of HS and, consequently, its influence on milk yield and characteristics [1]. Feed intake can establish certain blood metabolite alteration changes during HS. Besides those, metabolic profiling (known as metabolomics) has increasingly been used in clinical pharmacology and is an ideal tool for the acquisition of the several thousand metabolite alterations that are applied in the relationship between endogenous metabolite metabolism and body metabolism [9]. Metabolomics has been applied in cow investigations, in order to predict the risk of diseases [10], and has been used for biomarker and pathway discovery in some metabolic diseases in cows [10]. Given this review, metabolomics analyses provide a powerful platform for the identification of animals and humans associated with pathophysiological alterations resulting from exposures to specific environmental factors [10]. However, the metabolic changes related to short-term HS during the early lactation stage in dairy cattle still remain unclear. The activation process of the hypothalamus–pituitary–adrenal axis enhances the production and circulation of cortisol in the animal body. It is the primary indicator that ruminants can be identified when they are stressed [11]. Additionally, haptoglobin is one of the acute phase protein indicators commonly identified to animal health and inflammatory responses [11]. Hence, measuring these two correlated hormones in biological samples will allow better understating the mechanism behind the effect of short-term HS in dairy cows. HS response is a complex molecular process that involves the transcriptional and post-transcriptional regulation of stress-related genes. Acute environmental change initiates the heat stress response at the cellular level. Furthermore, microRNAs (miRNAs) was recognized as an important regulator of gene expression beyond the transcriptional stage and various biological reactions such as development, apoptosis, differentiation, and viral infection [12]. Additionally, miRNAs regulate numerous functions of bovine mammary epithelial cells (bMECs), which play a putative role in milk secretion [13]. In humans, it has been documented that miR-216 can regulate cell apoptosis through repressing target genes in several cancer cells [13]. Likewise in this study, we aimed at exploring the potential involvement of miR-216 in the milk of heat stressed cows. Furthermore, to better deal with the consequences of HS, we aimed to characterize the short-term effects of HS in terms of not only the alterations of productive performance characteristics but also the narrative response of metabolomics and gene expression.

Therefore, the objective of this study was to characterize short-term HS (4 day) in Holstein dairy cows using altered indicators of metabolomics, milk miRNA-216 and characteristics, and blood metabolites.

On the other hand, our traditional knowledge regarding HS effects is limited to long-term HS, in which the animals were housed outdoors or underwent uncontrolled environmental housing conditions, where the temperature and humidity were subject to many fluctuations over time, such as from being severe around noon to cooling in the evening. These phenomena may compromise our understanding of HS effects, due to disparities in environmental and housing situations, thus misleading the characterization of a framework of HS influence. In this regard, the outcome of this study provides insights towards better understanding how dairy cows exposed to chamber-controlled short-term HS can be characterized, not only through production performance parameters but also using newly presented indicators, such as metabolomics and miRNA analyses.

## 2. Materials and Methods

### 2.1. Experimental Design

This study was conducted at Konkuk University experimental farm, Republic of Korea. The experimental procedure was evaluated and approved by the Institutional Animal Care and Use Committee at Konkuk University (KU19121). Among the herd of multiparous early Holstein lactating cows, eight healthy animals with very similar conditions of days in milk (DIM = 40 ± 9 day; *p* > 0.05), milk production (milk yield = 30 ± 1.5 kg/day; *p* > 0.05) and parity (1.09 ± 0.05; *p* > 0.05) were chosen for this study. These criteria allowed homogeneity among the cows in the groups, and thus, any small changes could define reliably. The cows used for this specific experiment were assigned to two groups with distinct temperature and humidity index (THI) levels (of ~71 and ~86, respectively). The reason for choosing these two THI values (71 and 86) was that a THI of 71 represents the thermoneutral zone, while a THI of 86 represents severe HS; thus, we ensured that cows were under two distinct environmental conditions and not the range in between. As we had only four chambers (5 m × 5 m × 5 m), the two sets of THI levels were not conducted simultaneously. Hence, the first four chambers were used for the THI = 71 group, in order to provide the group with a low-humidity, low-temperature (LTLH) environment (period 1). Then, the second run was carried out with the other group of cows—the THI = 86 group—which were provided with a high-temperature, high-humidity (HTHH) environment (period 2). Each period, the environment inside the chamber was adjusted, in order to maintain the specified temperature and humidity for 3 days during the adaptation period. After the adaptation period, the temperature and humidity were controlled, for the cows to undergo HS for 4 days. The temperature was controlled using an air conditioner (CSVR-Q118E, Carrier corporation, Thailand), and the humidity was justified with the specified temperature and humidity using a humidifier (DE-9090UH, Zhongshan Xinhao Electrical.,LTD, Guangdong, China) and a dehumidifier (EDHA11W3, WINIA) throughout the experiment. Regarding Korean photoperiod conditions, light was provided from 09:00 h to 19:00 h. Experimental diets were fed twice daily, at 08:00 h and 14:00 h. All cows were fed common basal diets throughout the entire experimental period, according to NRC nutrient requirements (2001; Table 1). Basal diet and amino acid (AA) compositions are summarized in Table 1. Fresh water was provided five times daily during the experiment.

### 2.2. Temperature Humidity Index

The temperature humidity index (THI) was calculated using the following equation: (1.8 × T_db_ + 32) − [(0.55 − 0.0055 × RH) × (1.8 × T_db_ − 26)] [8,14], where T_db_ is the dry-bulb temperature (°C), and RH is the relative humidity (%). The temperature and humidity inside the chambers were controlled using an automatic computerized system. In the automatic chambers, the ambient temperature and humidity were fixed at 25 °C and 35–50%, respectively (low-temperature, low-humidity, LTLH), and 31 °C and 80–95%, respectively (high-temperature, high-humidity, HTHH).

### 2.3. Individual Animal Sampling and Analysis

#### 2.3.1. Feed and Water Intake

During the experiment, each cow was provided with feed and water twice a day (08:00 and 14:00 h) individually, while the remaining amount of feed (FI) and water (WI) intake were measured daily using a scale machine (GL-6000S Series, G-Tech International Co., LTD., Uijeongbu-si, Korea). 

#### 2.3.2. Milk Yield

Each early lactating cow was milked twice daily by portable machine (PMM 1B EPV170, Italy) at 05:00 and 17:00 h. The amount of the milk was recorded using a scale (GL-6000S Series, G-Tech International Co., LTD.) after each milking.

#### 2.3.3. Milk Composition Analysis

Daily milk samples (05:00 and 17:00 h) were mixed and sub-sampled bi-weekly for milk composition analyses. The milk was sampled in a 50 mL tube and analyzed immediately. The samples were analyzed using a milk scanner FT1 (Foss Alle 1 DK-3400 Hilleroed, Denmark) for protein, fat, lactose, solid-not-fat (SNF), somatic cells, milk urea nitrogen (MUN), acetone, beta-hydroxybutyrate (BHB), beta-casein, mono (MUFA)- and poly (PUFA)-unsaturated fatty acids, saturated fatty acids (SFA), total fatty acids (TFA), milk protein and fat yield, 3.5% fat-corrected milk (3.5% FCM), and energy-corrected milk (ECM). Milk protein and fat yields, 3.5% FCM, and ECM were calculated by multiplying the milk yield from protein and fat composition of the milk of an individual.

#### 2.3.4. Blood Profiles

Blood samples were harvested from the bovine jugular vein at 14:00 h on the 3rd day (adaptation period) and on the 7th day of the experiment (heat stress period). For blood hematology, analyses of white blood cell (WBC), lymphocyte (LYM), monocyte (MON), granulocyte (GRA), red blood cell (RBC), hemoglobin (HGB), hematocrit (HCT), mean corpuscular volume (MCV), red cell distribution width (RDWc), mean corpuscular hemoglobin (MCH), mean corpuscular hemoglobin concentration (MCHC), platelet count (PLT), mean platelet volume (MPV), plateletcrit (PCT), and platelet distribution width (PDWc) were conducted in ethylene-diamine-tetra-acetic acid (EDTA)-treated vacutainer (Becton-Dickinson, Franklin Lakes, NJ, USA) tubes, using an HM2 (VetScan HM2 Hematology System) machine. 

For the analysis of blood metabolites, the extracted blood samples from jugular venipuncture were transferred into non-heparinized vacutainers (BD Vacutainer, Plymouth, UK). Serum samples were obtained from blood after centrifugation at 2000× *g* for 15 min at 4 °C. The serum was isolated for analysis of metabolites, including albumin, blood urea nitrogen (BUN), calcium (CA), r-globulin, glucose (GLU), glutamic-oxalacetic transaminase (GOT), r-GT, magnesium (MG), non-esterified fatty acid (NEFA), inorganic phosphorous (IP), and total protein (TP). A total of 500 µL of serum from each sample was separated in a 1.5 mL tube (Eppendorf AG, Hamburg, Germany) and stored at −80 °C in a deep freezer, for further analysis using the analyzer (U9280-0002, Mississauga, NB, Canada). 

#### 2.3.5. Stress Hormone

The sample was extracted from the serum tube, separated by centrifuge, and 500 µL of the supernatant was sampled into each 1.5 mL tube (Eppendorf AG, Hamburg, Germany). The serum samples were analyzed using a cortisol ELISA kit (MBS701325, MyBioSource) and a haptoglobin ELISA kit (MBS739905, MyBioSource). The concentration of hormones, including cortisol and haptoglobin, in blood was measured using an analytical ELISA machine (PMT49984, BioTek Instruments Synergy Korea Ltd., Winooksi, VT, USA) measuring absorbance at a wavelength of 450 nm. The inter- and intra-assays of coefficients of variance for cortisol were 10% and 10%, while those for haptoglobin were 10% and 8%, respectively.

#### 2.3.6. Physiological Parameters

Heart rate (HR, bpm/min), skin temperature (ST, °C), and rectal temperature (RT, °C) were measured on the third and seventh days of each period, at 09:00 h. In order to measure skin temperature, a machine (S60, Caterpillar FLIR camera, Vernon Hills, IL, USA) was used to measure on the back, hips, and mammary glands. Rectal temperature measured using another machine (TES 1300, K Type thermometer, Taipei, Taiwan), which inserted into the rectum and held for 1 min. Heart rate was measured using a stethoscope on the neck side of each individual for 1 min.

#### 2.3.7. MicroRNA

Milk samples were transferred into five 50 mL tubes containing 0.5M EDTA at pH 8.0 (324503, AMRESCO, Bala Cynwyd, PA, USA), and incubated at 4 °C for 30 min. The samples were then centrifuged at 2700× *g* for 10 min at 4 °C. Thereby, the supernatant was removed from the skim milk layer. Afterwards, five 50 mL tubes were transferred into one 50 mL tube (12150, Taeshin Bio Science, Korea). After repeating the procedure three times, 1 mL of 1× PBS was added into the 50 mL tube and then filtered using a 200 µm (43-50200-03, Pluristrainer, Germany) membrane filter. Then, the tubes were centrifuged at 2700× *g* for 15 min at 4 °C. After removing the supernatant, 1× PBS was added and then transferred to a 2 mL tube. The milk sample was recovered and stored at −80 °C until further analysis. The isolation of miRNA from milk was performed using a mirVana^™^ miRNA isolation kit (AM1560, Thermo Fisher Scientific, Waltham, MA, USA), according to the manufacturer’s protocol. qPCR was performed using a TaqMan^™^ Fast Advanced Master Mix kit (4444557, Thermo Fisher Scientific). Thermal cycling was conducted according to the manufacturer’s recommended protocol, and all experiments were performed in duplicate. The TaqMan microRNA assays used in this study and their IDs were as follows: miR-216b (ID: 002326), miR-92a (ID: 000431).

#### 2.3.8. Metabolomics Analysis

##### Extraction of Serum Sample

Extraction of serum samples from blood was conducted by adding 450 µL of cold methanol to 150 µL of serum in an optimal ratio of 1:3. Each serum sample was homogenized (with a frequency of 30) using a mixer mill (Retsch GmbH & Co, Haan, Germany) for 10 min and stored at −20 °C for 1 h. After that, centrifugation of the sample was performed at 13,000 rpm for 10 min at 4 °C. The supernatants were then passed through an additional 0.2 µm PTFE filter and transferred to Eppendorf tubes. The supernatant was completely dried with a speed vacuum machine. The dried serum sample was dissolved by methanol and syringe filtration (0.2 µm), prior to GC-TOF-MS analysis. For the GC-TOF-MS analysis, two-step chemical induction was performed for the sample. First, an oximation process using the GC-MS analysis protocol was carried out with 50 µL of metoxyamine hydrochloride (20 mg/mL in pyridine, 90 min, 30 °C), followed by silylation using 50 µL of N-Methyl-N-(trimethylsilyl) trifluoroacetamide (MSTFA) (30 min, 37 °C). 

##### GC-TOF-MS Analysis

The GC-TOF-MS system was operated according to a previous study [15]. This was performed using an Agilent 7890B GC system (Agilent Technologies, Palo Alto, Santa Clara, CA, USA) and a Leco TOF Pegasus BT mass spectrometry (LECO, St. Joseph, MI, USA). A DB-5MS capillary column (30 m length, 0.25 mm i.d, 0.25 µm film thickness; J &W Scientific, Folsom, CA, USA) was used for helium gas flow of 1.5 mL/min. Afterwards, 1 µL of the sample derivatized using the GC-MS analysis protocol was injected into the split mode (1:10) for analysis. After 2 min of operation in an oven set to 75 °C, the temperature was increased to 300 °C, at a rate of 15 °C/min, and maintained for 3 min. To collect in electron ionization (EI) mode, mass data was collected using an EI method with ionization energy of 70 eV and a mass scan range of 50–600 m/z at an acquisition rate of 20 spectra/s. The ion source temperature and injector were set at 230 °C and 250 °C, respectively.

### 2.4. Statistical Analysis 

Pre-processing and statistical analysis of metabolomics data were carried out in accordance with a previous study [15]. Raw data collected through the GC-TOF-MS were converted to CDF (NetCDF) files with the LECO CROMA TOF software (version 4.44; LECO Corporation, accessed on 3 July 2020). For alignment, retention time correction, we used the online METALIGN software package (http://www.metalign.nl, accessed on 12 July 2020). Then, the final data were converted into an excel file. The data were placed in a three-dimensional matrix, utilizing information regarding peaks, peak areas, and sample names. The SIMCA-P+ software (version 12.0, Umetrics, Umea, Sweden, accessed on 20 July 2020) was used for multivariate statistical analysis. Unsupervised principal component analysis (PCA) was conducted, in order to investigate the general aggregate status and trends of other groups in all samples. After that, a supervised orthogonal partial least-squares discriminant analysis (OPLS-DA) model was used, in order to identify metabolomics that significantly differed, by maximizing the metabolomics changes between different groups. Variables were differentiated by variable importance in the projection (VIP) values. The statistical analyses were conducted based on the GLM procedure of SAS (Studio Version, SAS Institute Inc., Cary, NC, USA). All data were subjected to student’s *t*-tests (two-tailed) for comparisons between the means of the different groups. For the traits that were repeatedly measured, such as milk yield and behavioral data, the repeated measure analysis was performed, while the distribution of animals into treatments was considered as a random effect. Initial milk yield, compositions, and blood metabolites of the lactating cows showed no significant differences after the initial statistical analysis. Therefore, the continuous variable (covariate) was removed from the model. Analysis of covariance was utilized for correcting treatment means, controlling the experimental error, and increasing precision. Variance and covariance assumption structures, including AR(1), UN, CS, ANTE(1), TOEPH, ARH(1), and so on, were tested; the covariance structure that resulted in the lowest values for the Akaike information criteria was selected for the final analysis, due to its good fit to our design. Differences between the two subsets of data were considered statistically significant at *p*-values less than 0.05, while values between 0.05 and 0.10 were considered to indicate a significant trend tendency. 

## 3. Results

### 3.1. Feed and Water Intake

FI was significantly decreased (*p* < 0.05) by 10 kg/day in HTHH, compared to LTLH. Water intake showed no statistical difference (*p* > 0.10) between the two groups, but the HTHH group demonstrated a numerical increase of 14 kg/d (Table 2). 

### 3.2. Physiological Indicators

Rectal temperature showed a significant increase (*p* < 0.05) in the HTHH group, compared to the LTLH group. In addition, heart rate was higher (*p* < 0.05) in the HTHH group than in the LTLH group (Table 3). 

### 3.3. Blood Hematology and Metabolite Profile

Under HTHH treatment, blood RBC was tended to increase (*p* = 0.072). In contrast, the treatments did not influence WBC, LYM, MON, GRA, HGB, HCT, MCV, RDWc, MCH, MCHC, PLT, MPV, PLT, and PDWc in the blood (*p* > 0.10, Table 4).

Analysis of the blood metabolite profile in early lactating Holstein cows showed no effects of THI levels between the LTLH and HTHH groups, where GLU, NEFA, BUN, TP, albumin, r-globulin, CA, IP, MG, CHO, and GOT did not differ between the two groups (*p* > 0.10, Table 5).

### 3.4. Milk Yield and Compositions

#### 3.4.1. Milk Yield

Milk production was about 10 kg/day lower (*p* < 0.05) in the HTHH group, compared to that in the LTLH group (Table 6). 

#### 3.4.2. Milk Compositions

Under HTHH treatment, MFY, 3.5% FCM, and ECM all decreased (*p* < 0.05); however, MUN and MPY only tended to decrease (*p* > 0.05). In addition, there were no differences (*p* > 0.10) in protein, fat, lactose, SNF, somatic cells, acetone, BHB, beta-casein, MUFA, PUFA, SFA, and TFA between the two groups (Table 6).

### 3.5. Stress Hormones

We examined the stress hormone changes in the blood, including cortisol and haptoglobin levels, both of which were increased (*p* < 0.05) in the HTHH group, compared to the LTLH group (Table 7). 

### 3.6. MicroRNA Gene Expression

**Figure 1:**
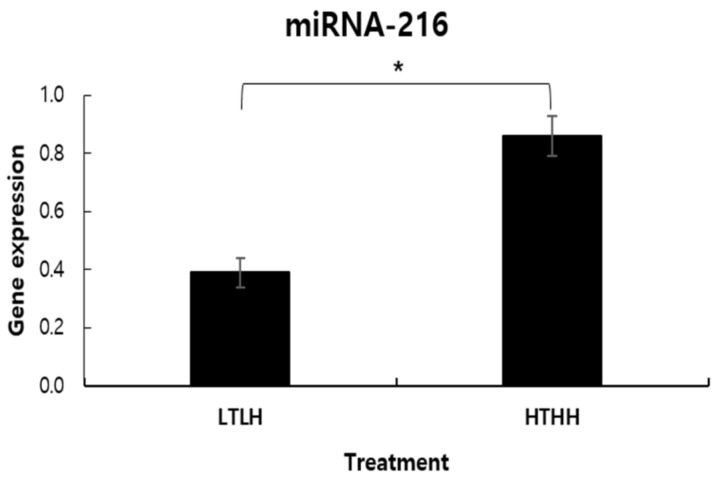
MicroRNA-216 alteration in dairy cows exposed to two distinct environmental conditions. Values (mean ± SEM) with asterisk (*) differ significantly, compared to the LTLH group (*p* < 0.05).

The abundance of miR-216 gene expression was affected by low- and high-temperature and humidity, being higher in the HTHH group (*p* < 0.05) than in the LTLH group (Figure 1). 

### 3.7. Metabolomics

**Figure 2:**
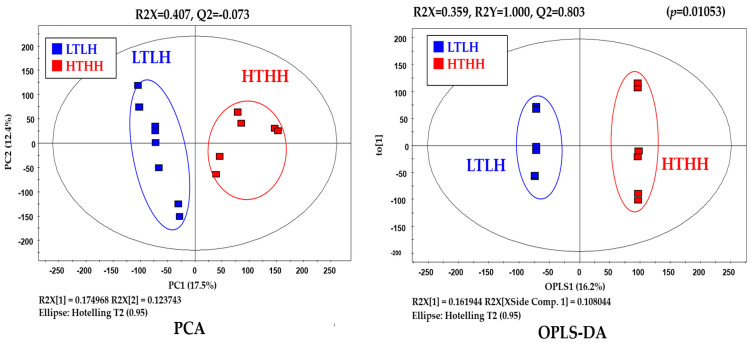
Blood metabolomics in dairy cows exposed to two distinct environmental conditions.

**Figure 3:**
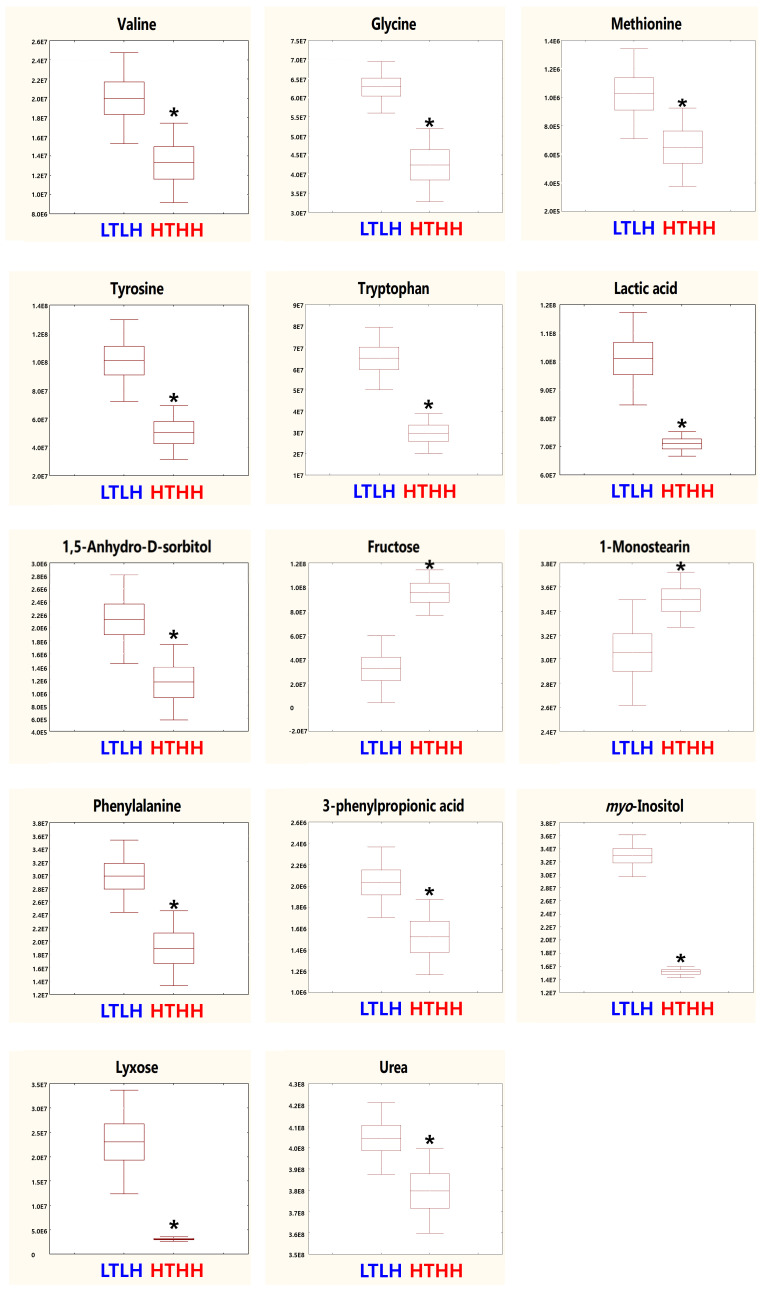
Metabolomics in dairy cows (*n* = 4) exposed to two distinct environmental conditions. Serum was assessed. LTLH vs. HTHH; VIP > 1.0, *p* < 0.05. Values (mean ± SEM) with asterisk (*) differ significantly, compared to the LTLH group (*p* < 0.05).

**Figure 4:**
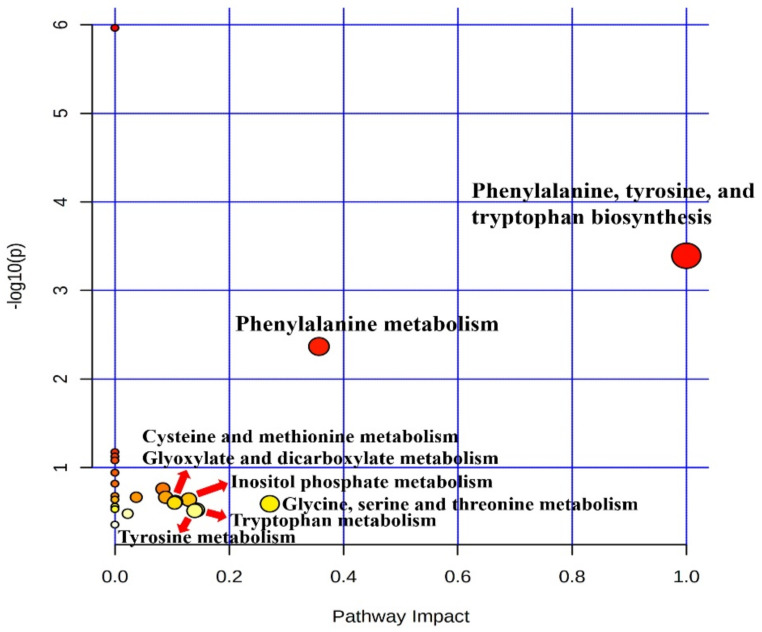
Metabolic pathway map related to metabolic profiling in serum, according to the effects of heat stress in the LTLH and HTHH groups.

Metabolic raw data of sera were collected for a multivariate statistical analysis. The status of metabolites in the LTLH or HTHH groups was determined in the score plot for PCA from serum (Figure 2). The distribution of metabolite parameters in serum (R^2^X = 0.359, R^2^Y = 1.000, Q^2^ = 0.803, *p* = 0.0105) showed a clear separation in the OPLS-DA model (Figure 2). According to the OPLS-DA analysis, potential metabolic markers were separated from the serum, based on a value of importance in the projection higher than 1.0 (VIP > 1.0). As a result, valine, methionine, phenylalanine, tyrosine, tryptophan, lactic acid, 3-phenylpropionic acid, 1,5-anhydro-D-sorbitol, myo-inositol, and urea were found to be decreased, while fructose and 1-monostearin were increased significantly (*p* < 0.05) in the HTHH group, compared with that in the LTLH group (Figure 3). For the pathway analysis, the online MetPa system (METABOANALYST 4.0, http://www.metaboanalyst.ca/, accessed on 16 January 2021) was used. Metabolites with significant changes were introduced into this online system, in an attempt to generate the metabolome view list. *Bos taurus* was selected for pathway analysis in the model organism interface. Targets were selected based on both impact value (not below 0.1) and p-value (no more than 0.05). Figure 4 shows the map regarding relevant metabolic pathways, while the function pathway results are presented in Table 8. As shown in Table 8, phenylalanine, tyrosine, and tryptophan biosynthesis (*p* < 0.001) and phenylalanine metabolism (*p* = 0.004) pathways were highly downregulated. 

## 4. Discussion

A decrease in FI is a very usual phenomenon in response to heat stress (HS) [1,4]. Under HS conditions, the amount of energy expended by the cow to maintain homeothermy increases (e.g., 20% more at 35 °C, compared to 20 °C). Panting also increases the maintenance requirement by 7–25% under HS. Therefore, the FI must increase to cover this additional energy cost [16]. However, during HS, FI decreases; this means that the energy status of the cow gets a double hit—greater energy costs to try to maintain homeothermy and lower energy intake [17]. In this regard, it is not surprising that milk production decreases. Indirectly, however, HS-decreased FI provokes consequences in connection with various physiological, metabolic, and blood parameters, in an attempt to lessen the effects of HS by activating homeostatic mechanisms across the body of the animal [5,8,18]. In this study, the mechanism behind the significant decrease in FI can indicate the direct effect of HTHH conditions on animals and the partial inability of cows to dissipate the excess of heat from their body, and thus, less intake will help them to reduce heat production (i.e., metabolic heat production and physically generated heat, known as heat increment) in their body. These results are in alignment with numerous previous studies that have claimed similar phenomena in response to HS [18]. 

Water intake has a high correlation with FI in animals and humans [1,5]. A normal phenomenon occurring in attempt to deal with HS is that cows tend to intake much more water than under normal conditions, in order to accommodate their body to dissipate heat by evaporation and by alterations in blood circulation. Additionally, under HS conditions, the cow loses water through its skin and respiration, in order to minimize the rise in body temperature [19]. Although HS may cause higher water consumption, on the other hand, decreased FI could also alleviate this water intake. In this study, HTHH did not seem to indicate the influence of FI in increasing water intake; however, water intake was partially induced by HTHH, showing a numerical increase. There is also the possibility that a larger sample size and longer period of HTHH exposure may have allowed us to observe a significant difference in water intake, which should be further studied. 

Several studies have shown that physiological indicators such as heart rate (HR) and rectal temperature (RT) are the foremost induced by short-term HS exposure in beef calves [8] and dairy cattle [20]. Kim et al. [8] have pinpointed that HR and RT are closely associated indicators, in response to HS, and are the most sensitive markers to be elevated. Therefore, HR and RT are likely to change in animals under HS, which is in agreement with the present results.

The body processes stressful information and elicits a response, depending on the degree of stress [21]. The body’s autonomic nervous system is broken down into the sympathetic nervous system (SNS) and the parasympathetic nervous system (PNS). In times of stress, the SNS is activated. The SNS is responsible for a cascade of hormonal and physiological responses [21]. The hypothalamus subsequently activates the SNS, and the adrenal glands release a surge of catecholamines, such as epinephrine [21]. This results in effects such as increased heart rate and respiratory rate. As the body continues to perceive stress, the hypothalamus activates the HPA axis. Cortisol is released from the adrenal cortex, which allows the body to continue to stay on high alert. Acutely, catabolic mechanisms of cortisol provide energy to the body [13]. The higher cortisol levels observed in HTHH group were in line with the higher heart rate in the corresponding group in this study. Kim et al. [8] provided a correlation study regarding the relationship between cortisol levels and blood and physiological parameters, including HR and RT. They mentioned that HR is correlated with the concentration of blood cortisol; as such, it has been utilized as an index for the regulation of animal body homeostasis [22]. Furthermore, it has been reported that the rise in blood pressure is associated with increased HR [23]. RT is also an important indicator for the homeostasis regulation of body temperature. The positive correlation between cortisol levels and RT may provide evidence that there exists a metabolic relationship between cortisol levels and RT. A previous study has suggested that the concentration of serum cortisol is a sensitive indicator of HS, and there is a significant correlation between cortisol and RT [24]. Therefore, the significant correlations between HR, RT, and the concentration of serum cortisol can be used to determine HS severity through physiological parameters in high-yielding dairy cows. Given the above review, the reasons behind increased HR and RT in the HTHH group, compared with the LTLH group, can be elucidated. 

Blood hematology encompasses several immune factors, including WBC, LYM, MON, and GRA, which are expected to be suppressed as a result of stress and an increase in cortisol, due to adverse correlations [25]. HS can cause immunosuppression in ruminants by inhibiting rumination [26], thereby leading to more chances of disease occurrence in the animals [25]. However, in the current study, most of the hematological parameters (except for RBC, which showed a decreased tendency) were not affected by short-term HS exposure. The unaffected parameters could be attributed to either the short time of HS exposure (only 4 day) [27,28] or low sample size in this study, due to natural fluctuations in blood parameters. In line with the present RBC results, a previous study has shown increases in the fraction of RBCs, including erythrocyte number, hematocrit value, and hemoglobin content, in HS group cows [29]. The tendency for higher RBC has been pinpointed, by Kumar and Pachauri [30], to be due to RBC release from the spleen and/or changes in erythrocyte stimulating factor (ESF) release, which is governed by the relationship between the oxygen demand of tissues and the amount of oxygen carried by the blood [30], in an attempt to dissipate heat from the body. In this study, the tendency of increase in RBC was also confirmed through higher serum haptoglobin, which we discuss, in detail, later (with respect to the hormonal effect on the HTHH group). On the contrary, Morar and Hutu [31] have reported that RBC, Hct, and Hb were decreased significantly in dairy cows under HS. Casella et al. [32] revealed the reduction in RBC, Hct, and Hb to be associated with a hemodilution effect due to increased water consumption in an attempt to facilitate evaporative cooling under HS [30]. However, we did not observe a significant increase in water intake in the HTHH group.

Blood metabolites may be directly (e.g., by FI [18]) or indirectly influenced by HS, in an attempt to reduce the deleterious effect of HS by activating body hemostasis mechanisms [8,18]. In this study, the serum metabolite profile, including GLU, NEFA, BUN, TP, ALB, r-GLU, Ca, P, MG, CHO, and GOT, showed no significant difference between two groups, LTLH and HTHH. As HS was accompanied with decreased FI, we expected to observe changes in some of the aforementioned profiles. However, contrary to our expectation, there were no changes in any of the metabolites. We could speculate some hypotheses behind this unchanged profile. One hypothesis could be the short application of HS, which may have compromised the ability of the body to challenge, with the first line of defense, against HS; while body lipolysis, gluconeogenesis, and other pathway activation or metabolite changes require time to elaborate [33]. Another hypothesis could be attributed to the low sample size (e.g., due to fluctuations in blood metabolites). It is well-known that blood metabolite alterations in response to stress conditions are subject to fluctuations, particularly when the size of the experimental unit is low [18]. In order to lessen these fluctuations, a larger sample size is recommended, in order to elaborate the variation effects within the data. In this way, we could possibly find significant differences in some blood metabolite profiles in future research. 

A natural phenomenon in response to HS is a decrease in FI (direct effect), where such a decrease may alternatively cause a decrease in milk yield and some of its characteristics (indirect effects of HS). One study has established that 35% of decrease in milk yield was due to decreased FI, whereas 65% was governed by the direct physiological effect of HS [4,25]. However, decreases in FI can be improved by feeding the cows early in the morning and at night. In these cooler periods of the day, cows can consume up to 80% of their total daily DM intake [16]. However, in cases where the night and morning times still exceed the upper critical THI (of 72)—such as the situation in this study, where cows were exposed to constant 24 h HS—the amount of feed consumed will not compensate for the greatly depressed intake during the day. Beyond THI = 69, each point increase in THI can cause milk reduction of 0.2 kg [1,25]. In other words, for each 1 °C raise in air temperature above the thermal comfort zone, an 0.85 kg decrease in FI occurs, which causes a milk yield decline of approximately 36% [1,4,25]. Given the above discussion, the significant decrease in milk yield of HTHH group in this study could be speculatively explained.

HS not only may lessen milk yield, but can also negatively affect milk constituents—particularly in high yielding dairy cows [25]. HS is widely responsible for a decline in milk fat, mainly due to higher concentrate ration and less fiber content or consumption of ration and, consequently, a disruption in fat synthesis in mammary glands due to increased body temperature [34]. The decline in milk protein content [13] may be due to specific down-thermoregulation activity of mammary protein synthesis [25]. These assumptions could explain the decline in milk fat and the tendency for decreased protein content in the HTHH group of this study. A raise in THI compromises the ability of dairy cows to dissipate excessive heat from the bodies [5], resulting in physiological changes such as reduced milk fat and protein contents [13,34]. Lower FI and thus less protein consumption aligned with decreased milk protein can also explain the decreased tendency of MUN. The mechanism may also rely on the lower urease activity in the wall of rumen and disruption in rumination [25,26], due to higher THI in HTHH group. Energy-corrected milk (ECM) determines the amount of energy in the milk, based upon milk fats and proteins, adjusted to 3.5% fat and 3.2% percent protein. Given the definition of ECM, it is obvious that the ECM showed a significant decrease in HTHH group, compared with the LTLH group, due to the lower milk fat and protein. The reasons for other milk constituents to remain unaffected in the HTHH group are unknown. 

Cortisol is the first hormone to look at in blood, saliva, or hair, when assessing stress situations, such as HS. Circulating cortisol has been shown to be a very sensitive index of heat stress, heralding the onset of poor tolerance of severe climates [5]. A high-temperature environment as a source of stress triggers a series of stress responses of the body [18]. The cortisol level adapts to the adverse environment, which is the evaluated index for the degree of stress and plays an extremely important role in the body [18]. Exposure to HS will shortly induce the production and release of cortisol from adrenal glands into the blood stream, in effect triggering a flood of glucose, which provides an immediate energy source for the body use. Given the above review, and as expected, the higher cortisol concentration in HTHH group was the result of short-term HS exposure in the corresponding animals. This result is consistent with numerous other studies, whether animals were exposed to short- or long-term HS [35,36]. It is worth noting that, as cortisol is one of primary responses of body to HS, it is not surprising to see its increase in less than an hour of stress exposure, and thus, cortisol increases can reflect acute stress conditions, which was observed under the conditions of this study. 

Haptoglobin is an acute phase protein produced by the liver, which the body uses to clear free hemoglobin (found outside of red blood cells) from circulation [8,22,35]. In other words, haptoglobin is a hemoglobin-binding protein which prevents oxidative damage by utilizing free hemoglobin and is integral in the formation of the haptoglobin–hemoglobin complex [36]. Hemoglobin is the iron-containing protein complex that transports oxygen throughout the body. It is normally found within red blood cells (RBCs) [35]. Haptoglobin binds to free hemoglobin in the blood. This forms a haptoglobin–hemoglobin complex, which is rapidly cleared out of circulation by the liver such that it can be broken down and the iron recycled. When an increased number of RBCs are damaged and/or break apart (hemolysis), they release their hemoglobin into the blood, increasing the amount of free hemoglobin in circulation, which is consistent with the obtained result of increased tendency of RBC, as hemoglobin carriers, in this study. On the other hand, when large numbers of RBCs are destroyed, haptoglobin levels in the blood will temporarily decrease, as the haptoglobin is used up faster than the liver can produce it [22,35]. A decrease in the amount of haptoglobin may be a sign of a condition that is causing red blood cells to be destroyed or to break apart [35,36], which was not the case in the present study. When the binding capacity of haptoglobin is exceeded, the free hemoglobin level in circulation goes up, which may cause tissue damage and/or organ dysfunction due to oxidative stress by free hemoglobin. In agreement with the presented results, an earlier study in beef calves showed higher haptoglobin supported by higher cortisol concentrations in blood [8]; in addition, another study [24] has reported an increase in serum haptoglobin in response to physical stress in cattle.

Thau et al. [21] explained the mechanism behind higher gene expression due to stress conditions as follows: Steroid hormones, such as cortisol, are primary messengers. They can cross the cytoplasmic membrane due to their fat-soluble properties. Cell membranes are composed of phospholipid bilayers, which prevent fat-insoluble molecules from passing through. Once cortisol passes through the cell membrane and enters into the cell, it binds to specific receptors in the cytoplasm. In the absence of cortisol, the glucocorticoid receptor binds to a heat shock protein (HSP) 90 chaperone protein in the cytosol. The binding of cortisol to the glucocorticoid receptor dissociates HSP90. The cortisol–receptor complex then enters the nucleus of the cell and affects gene transcription. In addition, MicroRNAs (miRNAs) are small single-stranded non-coding RNA, which repress post-transcriptional gene expression that can be altered by cortisol via targeting HSPs to modulate HS responses in dairy cattle [37]. Kumar et al. [38] reported increased miRNA expression due to summer HS in Tharparkar and Sahiwal cattle. Subsequently, Kishore et al. [39] reported higher expression of HSP40 transcript in Holstein Friesian, compared to Sahiwal cows, during summer HS [37]. After a short-term HS (2 h) exposure, Shandilya et al. [40] also reported induced mRNA expression of HSP40 and HPS70 in fibroblasts of zebu cattle. Taken together, the increase in miRNA expression in the HTHH group, compared to the LTLH group, in this study can be explained.

In our study, serum metabolic pathway analysis showed that the phenylalanine, tyrosine, and tryptophan biosynthesis and phenylalanine metabolism pathways were downregulated. Phenylalanine, tyrosine, and tryptophan are aromatic amino acids (AAA) which belong to the α-amino acid family for protein synthesis [41]. Phenylalanine, tyrosine, and tryptophan have been reported to play regulatory roles under heat stress, through their co-expression network [41]. In addition, AAAs play the role of precursors for numerous metabolomics related to protecting against stress, including melatonin, alkaloids, auxin, and phenolic compounds [41]. AAAs play important roles in the metabolic processes of microflora in all animal bodies. The AAAs and metabolites derived from them also play integral roles in the health of animals [41]. TAT1 is a T-system AA transporter, which plays an essential role in transporting AAAs. This transporter has been reported to be reduced in the chest and ileum of chickens affected by heat stress of 35 °C [42]. Phenylalanine had reduced levels in the brain and liver of chickens, as well as embryos, after exposure to heat stress of 38 °C. Phenylalanine is converted to a phenylamine neurotransmitter in response to the PLP-dependent aromatic enzyme decarboxylase [41]. In addition, it is often converted to tyrosine in the animal body, which is synthesized for epinephrine, dopamine, and norepinephrine neurotransmitters. Phenylalanine is also in charge of the biosynthesis of bacterial cell walls for inhibiting mureidomycins [43] and antibodies classes [44]. Tyrosine is changed to p-hydroxyphenylacetic acid by a mixture of bacteria and protozoa cultures, then converted to p-cresol [41], which plays an important role in the production of antioxidants [41]. Tyrosine is the precursor of the catecholamine neurotransmitters—dopamine and norepinephrine—which can administrate the behavioral, physiological, and neurochemical consequences, under a cold or heat stress environment, by adjusting the release of norepinephrine, thus demonstrating the role of tyrosine in protecting against the adverse effects of heat or cold stress [45]. In addition, when tyrosine is activated to its thiol ester form, it can be attached to the enzyme modular thiosteraease enzyme for use in antibiotic synthesis [41]. Tryptophan is the precursor for synthesis of serotonin, tryptamine, the neurohormone melatonin, and enzyme cofactors, which act as neurotransmitters [41]. A study in steers has shown that tryptophan supplementation can slowly increase in RT in response to acute heat stress through an increase in brain 5-HT, followed by a presumable increase in evaporative heat loss from the skin surface in cattle [46]. Among others, serotonin is involved in the melatonin synthesis metabolic process, which regulates growth activities in response to various biological stresses, such as pathogens, environmental toxins, and extreme temperature [46]. In a previous study, myo-inositol has been reported to be involved in glucose uptake and insulin signaling regulation, as well as adipogenesis regulation [9]. In the northern drosophila fly, high levels of myo-inositol were observed under a cold environment, which decreased in a warm or hot environment [47]. In our current study, we also found the myo-inositol level to be significantly lower in the HTHH group; however, we did not find any significant change in the pathway analysis. The accumulation of glycine betaine can reduce the effect of HS and improve productivity in lactating dairy cows [48]. A previous study has suggested that methionine supplementation can protect proteins from degradation by upregulating genes related to protein synthesis and decreasing genes related to protein breakdown [49]. In our current study, we observed the downregulation of serum glycine and methionine, which indicated that the heat stress in the HTHH group may have adverse effects in early lactating cows. However, from the pathway analysis, we did not find any change related to phenylalanine metabolism, inositol phosphate metabolism, glyoxylate and dicarboxylate metabolism, cysteine and methionine metabolism, glycine, serine and threonine metabolism, and tryptophan metabolism, which implies that these parameters had limited adjustment in the pathway analysis. As per the aforementioned review, the downregulation of these two pathways and their final impact metabolomics resulted in negative modulation in immune parameters and biological polymers (e.g., proteins, muscle cells, and so on) due to HTHH conditions, even when only exposed in the short-term (4 d). Thus, we postulate the importance of the negative association observed between HTHH and the final products of the resulting pathways in this study.

## 5. Conclusions

In this study, we successfully carried out metabolomics analyses in an attempt to characterize the influence of short-term HS on early lactating cows. According to the metabolic candidates, strong relationships with HTHH conditions were detected through the observed changes in the mentioned metabolic pathways. The results are worth further large scale investigation, in order to identify potential biological markers that can be used to accurately monitor HS conditions, as well as to develop a basis to further explain the physiological mechanisms underlying the metabolic pathway changes induced by HS.

## Figures and Tables

**Table 1:** Analyses of chemical compositions and amino acid profile of experimental feed.

Diets	TMR	Concentrate
Composition, % (DM)		
Dry matter	61.91	88.84
Crude Protein	6.06	18.06
Crude Fat	1.11	2.75
Crude Fiber	8.06	6.44
Crude Ash	3.15	6.17
Calcium	0.37	0.61
Phosphorus	0.17	0.50
Amino acids, % (DM)		
Tryptophan	0.06	0.19
Threonine	0.22	0.60
Serine	0.26	0.77
Proline	0.33	0.94
Valine	0.27	0.70
Isoleucine	0.17	0.51
Leucine	0.37	1.26
Tyrosine	0.10	0.39
Methionine	0.06	0.18
Cysteine	0.13	0.41
Lysine	0.25	0.52
Glycine	0.24	0.71
Alanine	0.30	0.84
Arginine	0.33	1.01
Glutamic acid	0.84	2.83
Aspartic acid	0.49	1.34
Histidine	0.10	0.33
Phenylalanine	0.22	0.68

TMR, total mixed ratio; DM, dry matter.

**Table 2:** Feed and water intake in dairy cows exposed to two distinct environmental conditions.

Treatment	LTLH	HTHH	SEM	*p*-Value
Parameters				
Feed intake, kg/day	40.10	30.11	1.980	0.001
Water intake, kg/day	99.67	113.76	5.080	0.183

LTLH: low-temperature, low-humidity (THI 71); HTHH: high-temperature, high-humidity (THI 86). THI = (1.8 × T_db_ + 32) − [(0.55 − 0.0055 × RH) × (1.8 × T_db_ − 26). SEM, standard error mean.

**Table 3:** Physiological parameters (rectal temperature and heart rate) in dairy cows exposed to two distinct environmental conditions.

Treatment	LTLH	HTHH	SEM	*p*-Value
Parameters				
Rectal temperature (°C)	38.33	39.13	0.161	0.001
Heart rate (bpm/min)	81.50	90.75	2.326	0.032

LTLH: low-temperature, low-humidity (THI 71); HTHH: high-temperature, high-humidity (THI 86). THI = (1.8 × T_db_ + 32) − [(0.55 − 0.0055 × RH) × (1.8 × T_db_ − 26). SEM, standard error mean.

**Table 4:** Blood hematological profile in dairy cows exposed to two distinct environmental conditions.

Treatment	LTLH	HTHH	SEM	*p*-Value
Parameters				
WBC, K/µL	10.52	10.45	0.822	0.970
LYM, K/µL	7.31	7.03	0.579	0.827
MON, K/µL	0.73	0.97	0.142	0.451
GRA, K/µL	2.48	2.46	0.432	0.984
RBC, M/µL	6.12	6.80	0.195	0.072
HGB, g/dL	10.18	10.75	0.347	0.451
HCT, %	28.18	30.06	0.891	0.328
MCV, fL	46.00	44.00	0.906	0.304
RDWc, %	20.48	20.40	0.211	0.875
MCH, pg	16.63	15.80	0.362	0.312
MCHC, g/dL	36.08	35.70	0.176	0.322
PLT, K/µL	467.25	592.25	43.592	0.165
MPV, fL	7.48	7.13	0.167	0.331
PCT, %	0.35	0.42	0.028	0.202
PDWc, %	32.73	32.20	0.643	0.715

LTLH: low-temperature, low-humidity (THI 71); HTHH: high-temperature, high-humidity (THI 86). THI = (1.8 × T_db_ + 32) − [(0.55 − 0.0055 × RH) × (1.8 × T_db_ − 26).Abbreviations: SEM, standard error mean; WBC, white blood cell; LYM, lymphocyte; MON, monocyte; GRA, granulocyte; RBC, red blood cell; HGB, hemoglobin; HCT, hematocrit; MCV, mean corpuscular volume; RDWc, red cell distribution width; MCH, mean corpuscular hemoglobin; MCHC, mean corpuscular hemoglobin concentration; PLT, platelet; MPV, mean platelet volume; PCT, plateletcrit; PDWc, platelet distribution width.

**Table 5:** Blood chemistry profile in dairy cows exposed to two distinct environmental conditions.

Treatment	LTLH	HTHH	SEM	*p*-Value
Parameters				
Glucose, mg/dL	58.75	68.75	3.432	0.157
NEFA, uEq/L	263.50	231.75	37.540	0.705
BUN, mg/dl	18.75	16.25	0.964	0.217
TP, g/dL	6.90	7.29	0.209	0.403
Albumin, g/dL	3.12	3.47	0.106	0.143
r-globulin, g/dL	3.78	3.82	0.164	0.897
CA, mg/dL	9.13	9.38	0.140	0.414
PI, mg/dL	6.13	5.25	0.295	0.149
MG, mg/dL	2.53	2.30	0.069	0.100
CHO, mg/dL	227.75	239.50	21.639	0.809
GOT, U/L	61.50	67.75	5.467	0.607

LTLH: low-temperature, low-humidity (THI 71); HTHH: high-temperature, high-humidity (THI 86). THI = (1.8 × T_db_ + 32) − [(0.55 − 0.0055 × RH) × (1.8 × T_db_ − 26). Abbreviations: SEM, standard error mean; NEFA, non-esterified fatty acid; BUN, blood urea nitrogen; TP, total protein; CA, calcium; PI, inorganic phosphorous; MG, magnesium; CHO, cholesterol; GOT, glutamic oxaloacetic transaminase.

**Table 6:** Milk yield and characteristic in dairy cows exposed to two distinct environmental conditions.

Treatment	LTLH	HTHH	SEM	*p*-Value
Parameters				
Milk yield, kg/d	40.63	30.89	2.293	0.016
Milk protein, %	2.86	2.94	0.037	0.360
Milk fat, %	4.13	3.68	0.341	0.551
Lactose, %	4.98	4.96	0.034	0.782
SNF, %	8.41	8.44	0.031	0.638
Somatic cells, 1000/mL	75.50	39.00	19.872	0.400
MUN, mg/dL	15.45	13.73	0.455	0.072
Acetone, mM	0.04	0.05	0.013	0.736
BHB, mM	0.08	0.07	0.006	0.473
Beta-casein, %	2.18	2.24	0.027	0.315
MUFA, %	1.44	1.28	0.119	0.562
PUFA, %	0.30	0.29	0.009	0.510
SFA, %	2.50	2.21	0.210	0.529
TFA, %	1.83	1.62	0.132	0.485
Milk protein yield, kg	1.17	0.91	0.065	0.099
Milk fat yield, kg	1.68	1.12	0.171	0.032
3.5% FCM	44.86	31.51	0.065	0.044
ECM	43.46	31.14	3.171	0.038

LTLH: low-temperature, low-humidity (THI 71); HTHH: high-temperature, high-humidity (THI 86). THI = (1.8 × T_db_ + 32) − [(0.55 − 0.0055 × RH) × (1.8 × T_db_ − 26).Abbreviations: SEM, standard error mean; SNF, solid-not-fat; MUN, milk urea nitrogen; BHB, beta-hydroxybutyrate; MUFA, mono-unsaturated fatty acid; PUFA, poly-unsaturated fatty acid; SFA, saturated fatty acid; TFA, total fatty acid; FCM, fat-corrected milk; ECM, energy-corrected milk.

**Table 7:** Blood cortisol and haptoglobin in dairy cows exposed to two distinct environmental conditions.

Treatment	LTLH	HTHH	SEM	*p*-Value
Parameters				
Cortisol	99.15	177.82	20.031	0.035
Haptoglobin	400.49	649.47	52.307	0.002

LTLH: low-temperature low-humidity (THI 71); HTHH: high-temperature high-humidity (THI 86). THI = (1.8 × T_db_ + 32) − [(0.55 − 0.0055 × RH) × (1.8 × T_db_ − 26).

**Table 8:** List of potential metabolic pathways that change in serum, according to heat stress.

Metabolite Pathway	*p*-Value	Impact	Metabolites
Phenylalanine, tyrosine, and tryptophan biosynthesis	<0.001	1.00	L-Phenylalanine L-Tyrosine
Phenylalanine metabolism	0.004	0.36	L-Phenylalanine
Inositol phosphate metabolism	0.230	0.13	Myo-Inositol
Glyoxylate and dicarboxylate metabolism	0.244	0.11	Glycine
Cysteine and methionine metabolism	0.250	0.10	Methionine
Glycine, serine, and threonine metabolism	0.257	0.27	Glycine
Tryptophan metabolism	0.302	0.14	L-Tryptophan

## Data Availability

Not applicable.
